# Supplementary material for: MetaRibo-Seq measures translation in microbiomes
Source: Nat Commun. 2020 Jun 29;11:3268. doi: 10.1038/s41467-020-17081-z (PMC7324362; doi:10.1038/s41467-020-17081-z)
Supplement: Supplementary file 10 — Supplementary Data 7 [file 41467_2020_17081_MOESM10_ESM.zip › File2/Confidence_High_Taxonomy/232427_out.krona.html]

Javascript must be enabled to view this page.

members
magnitude
magnitudeUnassigned
count
unassigned
taxon
rank

232427\_out

38

superkingdom
38
2

phylum
976
38

200643
38
class

order
38
171549

38
171550
family

239759
38
genus


SRS011239\_contig\_number\_19741SRS015663\_contig\_number\_27097SRS018623\_contig\_number\_2644SRS018936\_contig\_number\_12131SRS019445\_contig\_number\_9544SRS020394\_contig\_number\_4662SRS022137\_contig\_number\_19960SRS049402\_contig\_number\_16171SRS049896\_contig\_number\_6424SRS049995\_contig\_number\_26749SRS054352\_contig\_number\_23001SRS055017\_contig\_number\_contig-100\_279.157417SRS057480\_contig\_number\_7892SRS075773\_contig\_number\_28078SRS075878\_contig\_number\_2869SRS075984\_contig\_number\_21472SRS077849\_contig\_number\_24162SRS078242\_contig\_number\_2727SRS098061\_contig\_number\_10676SRS098881\_contig\_number\_16609SRS101376\_contig\_number\_12960SRS101433\_contig\_number\_14257SRS1041139\_contig\_number\_5091SRS104975\_contig\_number\_17498SRS140492\_contig\_number\_23447SRS142542\_contig\_number\_14405SRS142890\_contig\_number\_26115SRS143372\_contig\_number\_9582SRS143417\_contig\_number\_5176SRS143523\_contig\_number\_contig-100\_1021.138772SRS143895\_contig\_number\_7507SRS144135\_contig\_number\_35276SRS144362\_contig\_number\_32578SRS146812\_contig\_number\_47932SRS147346\_contig\_number\_40072SRS148721\_contig\_number\_41520SRS148817\_contig\_number\_6676SRS149075\_contig\_number\_3706
species
38
1118061
